# Hot-Melt and Pressure-Sensitive Adhesives Based on Styrene-Isoprene-Styrene Triblock Copolymer, Asphaltene/Resin Blend and Naphthenic Oil

**DOI:** 10.3390/polym14204296

**Published:** 2022-10-13

**Authors:** Sergey O. Ilyin, Viktoria Y. Melekhina, Anna V. Kostyuk, Nina M. Smirnova

**Affiliations:** A.V. Topchiev Institute of Petrochemical Synthesis, Russian Academy of Sciences, 29 Leninsky Prospect, 119991 Moscow, Russia

**Keywords:** pressure-sensitive adhesives, hot-melt adhesives, block copolymer, asphaltenes, rheology, adhesion

## Abstract

Asphaltene/resin blend (ARB) extracted from heavy crude oil was used to modify poly(styrene-block-isoprene-block-styrene) (SIS) to make it an adhesive. There were prepared double and triple mixtures containing 10–60% SIS, 10–40% ARB, and 10–50% naphthenic oil used as an additional plasticizer. The viscoelasticity of the mixtures at 25 °C and 120 °C was studied, their flow curves were obtained, and the temperature dependences of the loss tangent and the components of the complex modulus were measured. In addition, the mixtures were used as hot-melt adhesives (HMAs) and pressure-sensitive adhesives (PSAs) in the shear, peel, and pull-off tests of the adhesive bonds that they formed with steel. Both naphthenic oil and ARB act as plasticizers for SIS and make it sticky. However, only the combined use of ARB and the oil allows for achieving the best set of adhesive properties of the SIS-based mixture. High-quality HMA requires low oil content (optimal SIS/ARB/oil ratio is 50/40/10, pull-off adhesion strength (*τ*_t_) of 1990 kPa), whereas a lot of the oil is needed to give SIS characteristics of a PSA (SIS/ARB/oil is 20/40/40, *τ*_t_ of 100 kPa). At the same time, the resulting PSA can be used as a hot-melt pressure-sensitive adhesive (HMPSA) that has many times lower viscosity than HMA (13.9 Pa·s versus 2640 Pa·s at 120 °C and 1 s^−1^) but provides a less strong adhesive bond (*τ*_t_ of 960 kPa).

## 1. Introduction

Polymer adhesives can be divided into two types depending on how they form the adhesive bond: due to the change in chemical composition or during the course of physical processes. The first of them forms a permanent adhesive bond by means of procedures such as solvent evaporation [1,2] or chemical curing [3,4], which can be initiated by the influence of ultraviolet radiation [5,6] or a high temperature [7,8]. The second type of adhesive forms a bond in two cases: due to the application of pressure to the adhesive, which leads to wetting and filling the surface roughness of the glued bodies with it, or because of the temperature increase causing a reversible transition of the adhesive in the molten state. These adhesives are more environmentally friendly due to the absence of volatile components, residual reagents, and by-products in their composition.

Pressure-sensitive adhesives (PSAs) are a special class of materials that form adhesion joints with substrates under the action of small loads [9,10]. PSAs do not harden over time, do not dry out, have long-term vitality, and demonstrate adhesive properties when a small pressure is applied. They are widely used in everyday life, industry, and medicine [11,12]. To be a PSA, a polymer should combine sufficient (1) fluidity for the formation of an adhesive joint with high levels of cohesion strength and (2) elasticity [13], providing the strength of the joint. Thereby, viscoelasticity is the most important feature of PSAs, determining their properties [14,15,16].

Hot-melt adhesives (HMAs) are solvent-free fusible compositions based on thermoplastic polymers [17]. They form strong adhesive bonds between a wide range of materials at an elevated temperature and maintain the formed bonds after cooling [18,19]. HMAs are widely used in various fields, in particular, in the packaging, woodworking, footwear, and furniture industries [20,21,22,23]. They have a number of advantages, such as fast setting, environmental friendliness, economical usage, reduced fire hazard, the broad scope of application, resistance to moisture, long shelf life, good strength, and excellent adhesion characteristics.

Both PSAs and HMAs consist of a polymer base, a plasticizer, and a component that makes the system sticky (so-called tackifier) [24,25,26,27]. In terms of composition, the difference between PSAs and HMAs is quite arbitrary. Moreover, hot-melt pressure-sensitive adhesives (HMPSAs) have found application [12,28,29,30,31,32]. They differ from ordinary PSAs in both lower fluidity at high temperatures and greater rigidity at low ones. In addition, a feature of HMPSAs is the ability to form an adhesive bond under light pressure at room temperature, in contrast to conventional HMAs [33].

Many polymers are used as bases, the most common being ethylene-vinyl acetate, ethylene-acrylate, or ethylene-acrylic acid copolymers [34,35,36,37], different polyacrylates [38,39], polyisoprene [40], polyisobutylene [41], polyethylene [42], polyvinyl acetate [43], polylactide [44], polyamide [45], polyurethane [46,47,48], and polyester [49]. The polymer base represents more than half of the composition of adhesives and is responsible for their strength, elasticity, viscosity, and adhesion.

The second component in the composition of adhesives (about 30% of their volume) is resins, both natural (rosins, terpenes, and their derivatives) [50,51] and synthetic (hydrocarbon, terpene-phenol, and indene-coumarone) [52,53,54,55,56]. They are used to increase the adhesion of the compositions, improve the wetting of the glued surfaces, and lower viscosity [30].

The remaining about 20% of the adhesive compositions are various additives, such as waxes [18,57,58,59,60], plasticizers [61], pigments, fillers [62], and antioxidants [63]. Plasticizers are the most frequently used. They are introduced into polymer materials to improve plasticity during processing, increase the elasticity in the operation, and make the formulations cheaper. In addition, plasticizers facilitate the dispersion of the ingredients, reduce the forming temperature of the adhesive compositions, and improve their impact resistance under low temperatures; however, they sometimes decrease the softening point. Some plasticizers can enhance the flame, light, and heat resistance of polymers [64]. The most widely used plasticizers are esters, such as dibutyl phthalate, dioctyl phthalate, dioctyl sebacate, dioctyl adipate, trioctyl phosphate, and different trimellitates [65,66]. Chlorinated paraffins [67], mineral and vegetable oils [68], epoxidized oils [69,70], glycols [71], and other liquids are also used [72].

The polymer base for obtaining adhesives with an increased service life of adhesion joints is usually prepared from block copolymers, which are capable of forming adhesive joints due to soft fragments and are resistant to external loads because of microphase segregation and formation of a spatial structure from segregated hard blocks [73,74]. The most widely used block copolymers for producing adhesive compositions are triblock copolymers of styrene with either isoprene or butadiene [10,15,26,29,30,75]. However, these copolymers are characterized by relatively low energy of intermolecular interaction compared to the commonly used tackifiers, while the structure of the tackifier affects its miscibility with the copolymer blocks and, consequently, the properties of the resulting adhesive [28]. For obtaining improved adhesives based on styrene triblock copolymers, it is advisable to use hydrogenated resins (in particular hydrocarbon ones [76]) which are characterized by lower rigidity and softening points as compared with original resins [77].

Hydrocarbon (also known as petroleum) resins are obtained by polymerization of various fractions of crude oil and therefore they possess a wide molecular weight distribution and do not have a specific chemical structure [78,79]. However, natural compounds that are close in physical–chemical properties to hydrocarbon resins are present in heavy crude oil and bitumen. Such compounds are called asphaltenes [80], whose rheology is similar to that of conventional tackifiers and which, like them, can form miscible (at least partially) blends with certain polymers [81,82].

Nowadays, the problem of heavy crude oil transportation and refining has attracted global interest. A simple way to radically reduce the viscosity of heavy crude oil is by removing its highest molecular weight constituents, asphaltenes [83,84], but there arises the problem of their rational utilization. The use of asphaltenes for filling polymers is a new and promising method that is increasingly attracting the attention of researchers [85,86,87,88,89,90,91]. At the same time, due to the similarity of the properties of hydrocarbon resins and asphaltenes, the latter can act as a tackifier for polymer adhesive production. Moreover, asphaltenes do not need to be hydrogenated, since some methods of heavy crude oil deasphalting allow extracting resins (crude oil consists of saturates, aromatics, resins, and asphaltenes [92]) along with asphaltenes [93,94,95].

Resins are natural crude oil surfactants [96,97,98,99] that stabilize asphaltenes [100] and improve their interaction with various media, including polymer matrices [81]. However, the viscosity of resins and asphaltenes is quite high even in the molten state [95], so a plasticizer may still be required to create a polymer adhesive. Naphthenic oil is a rational choice, taking into account the miscibility of asphaltenes with cycloaliphatic compounds and their insolubility in linear alkanes [83,101]. However, neither the amount of asphaltene/resin blend required to make a polymer base sticky nor the necessary plasticizer content is known. On this basis, the present study was carried out using the typical styrene-isoprene-styrene triblock copolymer (SIS) as a polymer base to prepare multifunctional adhesives [102,103,104] based on its blends with asphaltenes, resins, and naphthenic oil. The microphase separation of styrene and isoprene blocks of this copolymer should act as physical cross-links. This eliminates the need for chemical curing of adhesives, which is an advantage because the formed adhesive bond can be destroyed, if necessary, and then restored from scratch. In the first step, we will examine how oil and asphaltenes with resins affect the rheological and adhesion properties of SIS separately. In the second step, we will determine the optimal concentration of asphaltenes and resins to achieve the best properties of the resulting adhesive, while in the third step we will do the same with the SIS/oil ratio. In all cases, we will consider the adhesives for their application both as HMAs and as PSAs.

## 2. Materials and Methods

### 2.1. Materials

Styrene-isoprene-styrene triblock copolymer Vector 4411A (Dexco, San Diego, CA, USA) containing 44 wt% styrene units and having a number-average molecular weight of 82,000 g/mol, a dispersity of 1.05, and a melt flow index of 40 g/10 min (200 °C, 5 kg load) was used as a polymer base. It was plasticized by refined naphthenic oil Shellflex 371 (Equilon Enterprises LLC, Houston, TX, USA) with a glass transition temperature of −64 °C, a pour point of −34 °C, and a density of 0.901 g/mL. Asphaltene/resin blend (ARB) was obtained by adding a 15-fold excess of hexamethyldisiloxane (ECOS-1, Moscow, Russia) to heavy crude oil (Ashalchinskaya oilfield [105,106], Almetyevsk, Russia) as described earlier [107]. The weight average molecular weight, dispersity, and softening point of ARB were 828 g/mol, 1.14, and 71 °C, respectively. ARB consisted of 19.3% heptane-insoluble asphaltenes and 49.7% resins, while the rest were admixtures of heavy aromatic compounds and crystallizing saturates [95].

### 2.2. Blend Preparation

Two series of triple SIS/ARB/oil mixtures were considered. In the first series, the ratios between SIS and naphthenic oil were equal (50/50, 45/45, 40/40, 35/35, and 30/30 wt%/wt%), while the ARB content was varied (from 0 to 40 wt%, i.e., to the maximum tackifier concentration commonly used in practice [76,108]). In the second series, the ratio between SIS and oil was changed (10/50, 20/40, 30/30, 40/20, and 50/10 wt%/wt%), while the ARB concentration was 40 wt% (the concentration appeared to be the best from the test results of the first series). In addition, binary SIS blends containing 40% of naphthenic oil or ARB were prepared for comparison of their role on SIS properties. In all cases, the components were compounded using a PolyDrive twin-rotor laboratory mixer (HAAKE, Vreden, Germany) equipped with sigma-shaped rotors at 120 °C for 20 min under a rotor rotation speed of 60 rpm. The films for studying the adhesive and strength properties were shaped with an HLCL-1000 laminator (ChemInstruments, West Chester Township, OH, USA) between two siliconized polyethylene terephthalate films at 120 °C. The thickness of the adhesive films being formed was 160 ± 20 μm. There was not any curing of the samples.

### 2.3. Methods

The rheological properties were studied at 25 °C and 120 °C with a Discovery HR-2 rotary stress-controlled rheometer (TA Instruments, New Castle, DE, USA) using a cone-plate working unit with a cone diameter of 25 mm and an angle between the cone generatrix and the plate of 2°. The flow curves were obtained in steady-state mode by stepwise increasing the shear rate from 0.001 s^−1^ to 100 s^−1^ and plotting the viscosity (*η*) versus shear stress (*σ*) dependences. The frequency dependences of storage (*G*′) and loss (*G*″) moduli were determined in the linear viscoelasticity region by varying the angular frequency (*ω*) in the range 0.0628–628 rad·s^–1^. The temperature dependences of the storage and loss moduli were measured at an angular frequency of 6.28 rad·s^–1^, a strain amplitude of 0.1%, and a temperature decrease rate of 5 °C/min. The equations for calculating the rheological characteristics can be found elsewhere [109,110], the relative error of their determination did not exceed 5%.

The obtained SIS/ARB/oil mixtures were evaluated in terms of their use as both hot melts and pressure-sensitive adhesives. The adhesion properties for HMAs were studied by the tensile and lap shear tests, while for PSAs: by the peel test at an angle of 90° and the probe tack method.

The tensile adhesion tests for HMAs were performed with a TA.XT plus device (Stable Microsystems, Goulford, CT, USA). A steel cylindrical rod (4 mm in diameter, mean surface roughness of 500 nm) was lowered to contact with the adhesive layer at a velocity of 0.5 mm·s^−1^ and was pressed against the sample with a force of 500 g for 900 s at 120 °C. Then, the temperature was reduced to 25 °C and after keeping for 900 s, the rod was withdrawn at a velocity of 0.1 mm·s^–1^; the force arising in the process was determined, and the pull-off strength (*τ*_t,HMA_) was calculated.

The lap shear adhesion strength for HMAs (*τ*_s,HMA_) was evaluated using a TT-1100 tensile tester (ChemInstruments, West Chester Township, OH, USA) at a crosshead motion velocity of 0.63 mm·s^−1^ and a temperature of 25 °C. Two galvanized steel plates with a width of 10 mm and a thickness of 250 μm were glued together by the mixture under study at 120 °C with the formation of an overlap; the gluing area was 10 mm × 5 mm. The plates were pressed to each other with a force of 1600 g for 1 min, then cooled, unloaded, and tested.

The resistance to peel at an angle of 90° for PSAs (*τ*_p,PSA_) was evaluated in accordance with FINAT FTM 2 standard using a TT-1100 tensile tester at a pulling velocity of 5.1 mm·s^–1^ and a temperature of 25 °C. Prior to testing, the adhesive specimen 10 cm long and 12.5 mm wide was glued to polished steel plate and rolled two times with a standard 2 kg roller. Then, the mean peel force was determined.

Adhesion probing studies of PSAs were performed with a TA.XT plus device at 25 °C. A film being tested was transferred onto a glass support, and a steel cylindrical rod (9.94 mm in diameter, mean surface roughness of 500 nm) came down to contact with the sample at a velocity of 0.5 mm·s^−1^ and pressed on the adhesive with a force of 500 g for 10 s. Then, the rod was withdrawn at a velocity of 0.1 mm·s^–1^, the force arising in the process was determined, and the pull-off strength (*τ*_t,PSA_) was estimated.

The mechanical strength at break of the adhesive films (*τ*_c_) with a width of 5 mm, a length of 30 mm, and a thickness of 160 μm were measured using a TT-1100 tensile machine at a temperature of 25 °C and a crosshead motion velocity of 0.63 mm·s^–1^. In all cases of adhesion and strength tests, at least 7 specimens in a series were used.

## 3. Results

### 3.1. The Effect of Naphthenic Oil and ARB on SIS Properties

**Figure 1:**
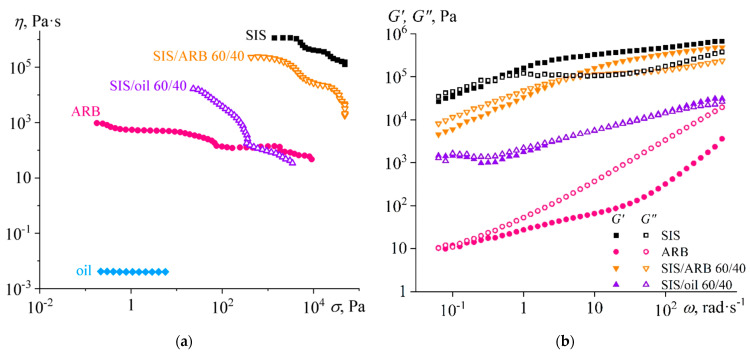
Flow curves (**a**) and frequency dependences (**b**) of storage (filled symbols) and loss (hollow symbols) moduli for styrene-isoprene-styrene triblock copolymer (SIS), asphaltene/resin blend (ARB), naphthenic oil, and their binary mixtures at 120 °C. Figure 1b shows no data for the oil because it does not exhibit viscoelasticity.

At an adhesive mixing temperature of 120 °C, SIS and ARB melts are non-Newtonian fluids (Figure 1a). Their viscosity decreases with increasing shear stress, and the ARB viscosity is 3–3.5 decimal orders less than the SIS viscosity. The oil viscosity does not depend on the shear stress and is 8.5 decimal orders less than the SIS viscosity. The combination of the block copolymer with oil or ARB (in such a way that there is more than half of the polymer base) expectably leads to a decrease in the viscosity, and the oil reduces the viscosity more efficiently.

When mixing two liquids, the blend viscosity often obeys the logarithmic additivity rule, according to which the logarithm of the blend viscosity is the weighted average of the logarithms of the component viscosities [111,112]:

log*η*_12_ = *φ*_1_ log*η*_1_ + *φ*_2_ log*η*_2_, (1)

where *φ*_1_ and *φ*_2_ are the volume fractions of the components, while *η*_12_, *η*_1_, and *η*_2_ are the viscosities of a binary mixture and its first and second components, respectively. In the case of blending polymers, this relation may not be performed if the components are immiscible, or the rheological properties of the mixture depend on its morphology [113,114].

The measured low-shear viscosity of SIS blended with 40 wt% of ARB is 10^5.37^ Pa·s (at 10^−3^ s^−1^ and 120 °C), while the calculated viscosity for this blend is 10^4.74^ Pa·s. In turn, the experimental and calculated viscosities of SIS containing 40 wt% of naphthenic oil are 10^4.22^ Pa·s and 10^2.69^ Pa·s, respectively. Thus, the mixture viscosity does not obey the logarithmic additivity rule, which is probably due to the microphase separation of the block copolymer and the concentration of the introduced oil, asphaltenes, and resins in one of the microphases.

Since the melt viscosity of a microphase-separated block copolymer is determined mainly by its rigid blocks [115] and there is a positive deviation from the logarithmic additivity rule (the viscosity decreases weakly), it can be assumed that the oil and ARB resins are to some extent more strongly concentrated in the microphase of isoprene blocks. This is also confirmed by changes in the SIS viscoelastic properties (Figure 1b), which are important since they determine the adhesion performance of PSAs [116].

There can be distinguished two regions in the frequency dependences of the storage and loss moduli of the SIS melt. In the region of low frequencies (*ω* < 1 rad/s), the loss modulus exceeds the storage one, i.e., SIS behaves more like a liquid and is able to function as a hot melt adhesive at sufficiently long observation times. In the high frequency region, SIS pass into the glassy state (due to the proximity of the test temperature to the glass transition temperature of polystyrene blocks, which is 75 °C [81]) and loses its ability to spread and stick under short-term exposure (less than 1 s).

Naphthenic oil does not exhibit viscoelasticity and its addition to SIS qualitatively changes the nature of the melt viscoelastic properties. Firstly, there is an increase in the frequency dependence of the storage and loss moduli in the high frequency region, i.e., the region of transition to the glassy state becomes less pronounced and more extended in frequency. This is due to the expansion of the relaxation time spectrum of polystyrene segments, which may be associated with partial plasticization of polystyrene blocks and an increase in their miscibility with polyisoprene blocks under the influence of oil. Secondly, the storage and loss moduli are comparable in magnitude in the low frequency region and practically do not depend on the frequency. Such behavior in the low frequency region is associated with the structuring of systems [117,118,119], in this case with the microphase segregation of polystyrene blocks that are in a highly elastic (rubbery-like) state. In other words, the introduction of oil into the SIS does not lead to the disappearance of the microphase separation, but it becomes more noticeable due to the shift of the glass transition region towards higher frequencies and lower temperatures.

The ARB melt behaves more like a liquid (*G*” > *G*’) in a wide frequency range. However, it is not an ordinary viscoelastic liquid, because its behavior in the low-frequency region does not correspond to the Maxwell model when *G*’ ~ *ω*^2^ and *G*” ~ *ω* [109]. The dependences of the storage and loss moduli have lower slopes, which can be associated with the structuring of asphaltenes, e.g., lower slopes of *G*’(*ω*) and *G*”(*ω*) are characteristic of heavy crude oils and bitumen containing a high concentration of asphaltenes [120,121]. The addition of ARB into the SIS melt does not change the viscoelastic properties as much as it did in the case of the addition of naphthenic oil. In fact, there is only a moderate quantitative decrease in the storage and loss moduli: a shift of their frequency dependences to the high-frequency region. In other words, asphaltenes and resins slightly plasticize the microphase of polystyrene blocks, leading to an increase in the angular frequency (and consequently a decrease in temperature) at which glass transition occurs (e.g., when *G*’ = *G*”).

Meanwhile, the introduction of ARB does not reduce the SIS cohesive strength but rather slightly increases it (*τ*_c_, Table 1, a note to the table contains statistical analysis data). This can be related to the fact that resins and asphaltenes pass into a glassy state upon cooling down to 25 °C and no longer plasticize the block copolymer; moreover, asphaltenes start to play the role of reinforcing particles [81,82]. In contrast, the addition of the same amount of oil (40%) to SIS expectably reduces its tensile strength by three times.

Actually, SIS is able to act as a hot melt adhesive and forms a relatively strong bond to the steel surface (Table 1). The introduction of asphaltenes and resins to the SIS does not noticeably change the adhesive properties of the hot melt adhesive during shear bank (*τ*_s,HMA_) and pull-off (*τ*_t,HMA_) tests of the adhesive joints (*p* > 0.1, see the note to Table 1). However, the adhesive shear strength is reduced by three times in the case of adding oil (*p* < 0.01), which indicates the cohesive nature of the adhesive joint destruction under this character of the load. The latter is indirectly confirmed by the fact that the addition of oil to the SIS does not affect the adhesive strength during the pull-off test (*p* > 0.1).

The pure SIS is not sticky at 25 °C and is unable to function as a PSA. The addition of asphaltenes or oil to SIS makes it slightly sticky because of plasticization, but the strength of the resulting joints is rather low. In this case, the mixture with asphaltenes works better at high rates of application and detachment of the adhesive (i.e., during peel tests, *τ*_p,PSA_) but badly at slow rates (during tensile tests, *τ*_t,PSA_). This can be explained by the viscoelasticity of this mixture: its storage modulus is higher than the loss modulus at high frequencies, while at low frequencies the ratio between the moduli is reversed (see Figure 1b). In turn, the mixture with oil has lower storage and loss moduli, which leads to poorer adhesion characteristics at high detachment rates of this adhesive when it peels off. However, this mixture performs better at slow detachment rates of probe tack tests than the mixture with asphaltenes, which is due to the comparability of its storage and loss moduli to each other even at low frequencies.

Thus, neither SIS nor oil improves adhesion of the SIS melt (more precisely, its ability to wet the surface). However, they slightly enhance SIS adhesion at normal temperature. In the latter case, the oil improves adhesion due to plasticization of the SIS, changing its viscoelastic properties and imparting stickiness. ARB does not noticeably plasticize the SIS at normal temperature and therefore leads to very limited improvement in stickiness. In any case, ARB and oil separately do not significantly improve the adhesion characteristics of the block copolymer. Let us see if they can make SIS a better adhesive by acting together.

### 3.2. The Effect of the ARB Concentration on the Adhesive Properties

**Figure 2:**
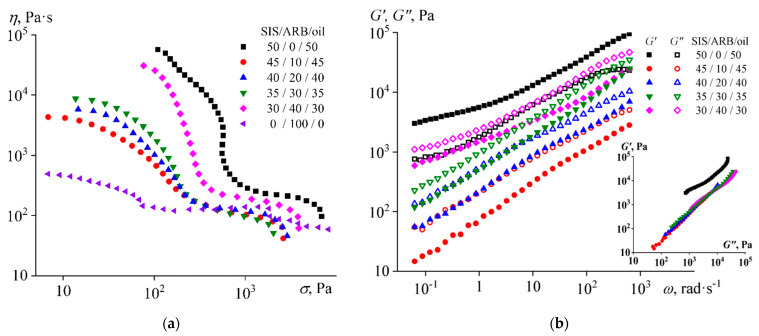
Flow curves (**a**) and frequency dependences of storage and loss moduli (**b**) for triple mixtures containing different concentration of asphaltene/resin blend (ARB) and an equal ratio between styrene-isoprene-styrene triblock copolymer (SIS) and naphthenic oil at 120 °C. The insert shows the Cole–Cole plots.

The flow curves of the melted mixtures with different concentrations of asphaltene/resin blend and an equal ratio between SIS and naphthenic oil are shown in Figure 2a. The high oil content in the block copolymer leads to an increase in its non-Newtonian behavior. As a result, a vertical section of constant shear stress of about 560 Pa can be identified on the flow curve of the SIS/oil mixture. This section can be interpreted as the yield stress of the mixture, i.e., stress causing the destruction of the spatial structure that gives the sample its solid-like properties [123]. In our case, this structure is formed by a continuous microphase from the rigid polystyrene blocks.

The addition of 10% of ARB to the binary SIS/oil mixture reduces the effective viscosity in the entire range of shear stresses, so that the viscosity of the mixture at high stresses becomes comparable to that of ARB. Thus, ARB plasticizes at least the polystyrene microphase, which determines the value of the copolymer effective viscosity at low shear stresses. In addition, ARB possibly improves the compatibility between polystyrene and polyisoprene microphases, resulting in lower yield stress. Nevertheless, a higher ARB content of about 20–30% does not lead to a further decrease in viscosity but conversely increases it in the region of low shear stresses. The increase in effective viscosity becomes even more pronounced with the addition of 40% ARB, although the viscosity still does not reach the level characteristic of the original SIS/oil mixture.

Thus, ARB acts as a SIS plasticizer but is only effective at low concentrations. The effect of plasticization can also be found in the study of the melt viscoelastic properties (Figure 2b). The storage modulus exceeds the loss modulus in the entire frequency region for the SIS/oil mixture, but the addition of 10% of ARB reverses the situation (making *G*” > *G*’) and significantly reduces both moduli by 1–2 decimal orders. Higher ARB concentrations increase the moduli but do not fundamentally change the pattern of the viscoelasticity of the systems. The latter becomes apparent when plotting Cole–Cole plots (the insert in Figure 2b). All plots for mixtures containing ARB are superimposed on each other, which means their identical microstructure [124,125].

**Figure 3:**
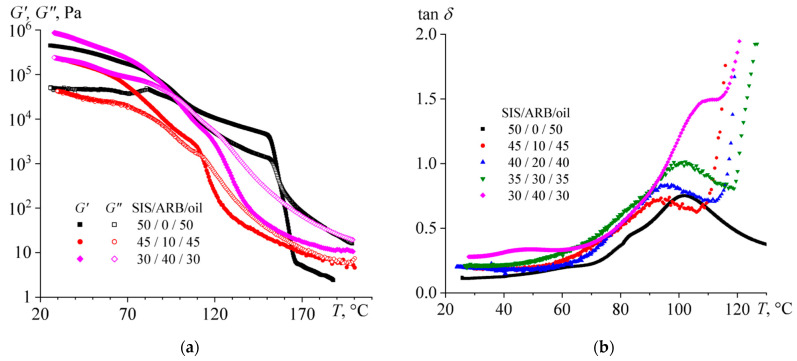
Temperature dependences of (**a**) storage and loss moduli and (**b**) loss tangent for triple mixtures containing different concentration of asphaltene/resin blend (ARB) and an equal ratio between styrene-isoprene-styrene triblock copolymer (SIS) and naphthenic oil.

The mixtures undergo structural transformations with varying temperature, which is reflected in the values of their storage and loss moduli (Figure 3a). The transition from liquid- to solid-like behavior (when *G*’ = *G*”) can be distinguished for the SIS/oil mixture at 157 °C. The transition can be associated with the microphase separation of this mixture during its cooling, because of which rigid blocks of polystyrene form a continuous phase. With the introduction of 10% of ARB, the sharp transition from liquid-like to solid-like behavior disappears, and the point of equality of the storage and loss moduli shifts towards lower temperatures. Thereby, ARB suppresses microphase separation to some extent, as well as plasticizing the mixture, since its moduli are reduced.

The plasticization of the mixture is also confirmed by a decrease in the glass transition temperature of polystyrene blocks, which can be determined from the position of the local maximum of the loss tangent (Figure 3b). With the introduction of 10% of ARB to the SIS/oil mixture, the glass transition temperature decreases from 101.9 °C to 94.2 °C. At the same time, a higher ARB concentration slightly increases the glass transition temperature but enhances the absolute value of the loss tangent, so that it becomes greater than 1.0 at the ARB content of 40%. This means that at a high concentration of ARB, the mixture is able to retain its liquid-like behavior down to lower temperatures and act better as a pressure-sensitive adhesive.

**Figure 4:**
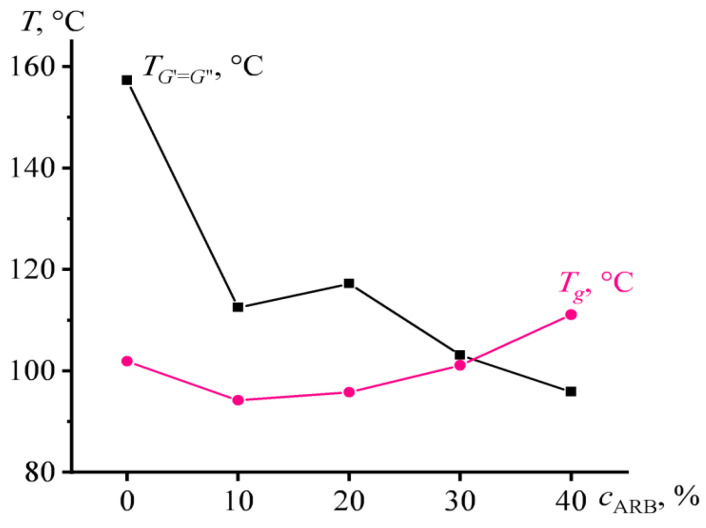
Concentration dependences of glass transition temperature and liquid–solid transition point for mixtures with different concentrations of asphaltene/resin blend (ARB) and an equal ratio between styrene-isoprene-styrene triblock copolymer and naphthenic oil.

The liquid–solid transition temperature (when *G*’ = *G*”) determines the temperature at which the mixture can be used as HMA. This temperature decreases significantly with increasing ARB concentration, despite the fact that at the same time the glass transition temperature passes through a local minimum and then increases (Figure 4). Thereby, these two temperatures are not directly related. The liquid–solid transition is connected not only with the glass transition of the polystyrene microphase but also with the microphase separation itself, which is suppressed by ARB.

**Figure 5:**
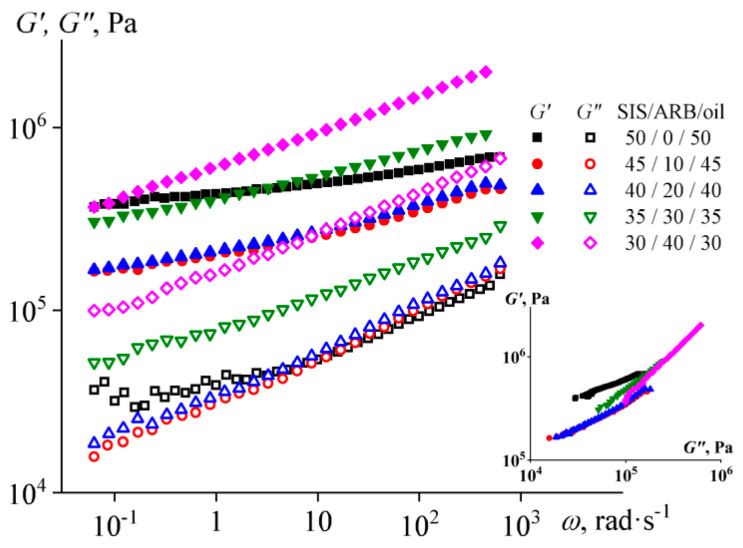
Frequency dependences of storage and loss moduli for triple mixtures containing different concentration of asphaltene/resin blend (ARB) and an equal ratio between styrene-isoprene-styrene triblock copolymer (SIS) and naphthenic oil at 25 °C. The insert shows the Cole–Cole plots.

It is interesting to note that the temperature dependence of the loss tangent shows a local maximum at 47 °C for the mixture containing 40% of ARB (Figure 3b). This temperature is close to the glass transition temperature of ARB, which is 50–70 °C [95]. This may mean that a part of the ARB in the mixture is present as a separate phase. The latter is confirmed by considering the frequency dependences of the storage and loss moduli of the mixtures at 25 °C in the coordinates of the Cole–Cole plot (the insert in Figure 5). The mixtures containing 10–20% and 30–40% of ARB have different microstructures because their Cole–Cole plots do not overlap each other. At the same time, there was no such mismatch in Cole–Cole plots at high temperatures (see the insert in Figure 2b). Thus, the structure of all ternary blends at high temperatures is the same and ARB acts as an SIS plasticizer. However, when mixtures containing 30–40% ARB are cooled, phase separation occurs with the release of an ARB part into a separate phase. Because of the ARB release, the plasticization of the SIS is reduced, and the ARB particles act as reinforcing ones. As a result, the mixture containing 40% ARB is more rigid than the original ARB-free mixture (Figure 5).

With the introduction of ARB, the cohesive strength of the mixture passes through a minimum corresponding to the composition with 20–30% of ARB (Table 2). The strength reduction is probably related to the plasticization of the mixture, which occurs due to the dissolution of the low molecular weight resins of ARB in the SIS. In the case of a high ARB content, the strength of the mixture conversely increases, which can be associated with the reinforcing effect of asphaltenes that do not dissolve in the polymer medium at their high content and act as a reinforcing phase. A similar situation was observed with epoxy resin, when the strength of the cured composite also passed through a minimum with increasing ARB content [82].

The addition of ARB significantly improves the adhesive characteristics of the SIS/oil mixture. The original mixture containing SIS and oil in the ratio of 1:1 is not able to play either the role of HMA or the role of PSA (Table 2). This is probably due to the too-high oil content, since at its lower concentration (40%, see Table 1) the binary mixture could at least act as a hot melt adhesive.

The addition of 10% ARB makes it possible to use the mixture as PSA: the peel strength of the adhesive joints takes a non-zero value (Table 2). The ability to act as a PSA is apparently associated with the plasticizing action of the ARB resins, which leads to a decrease in the glass and liquid–solid transition temperatures of the mixture (see Figure 4). At the same time, the addition of 10% ARB is not enough to give the mixture the role of HMA. The situation changes with the introduction of a larger content of ARB. Moreover, the more the ARB content, the higher the shear and pull-off strength of the adhesive joints of the hot melt adhesive (and the data are becoming more statistically significant, see the note to Table 2). This may be due to both an increase in the cohesive strength of the mixture and an improvement in the wetting of the substrate by the adhesive when ARB is added to it [81]. However, the nature of the joint destruction is adhesive and the increase in the cohesive strength of the adhesive cannot be the main reason for improving its adhesive properties.

When the content of ARB is 40%, the strength of the adhesive joints is maximal, especially when the mixture is used as HMA (*p* < 0.05). Therefore, it is reasonable to take this concentration as the optimal one. Lower ARB concentrations reduce adhesion, while higher ARB contents are impossible to introduce into the SIS due to poor mixing of the components and the inhomogeneous composition of the resulting blends. The latter is probably due to the immiscibility of the components in such blends and the formation of a continuous phase from ARB, the viscosity of which is much lower than the viscosity of the SIS. At the same time, the strength of the joints formed at 25 °C when using the mixture as PSA is not maximal in the case of 40% ARB concentration. It is reasonable to assume that an improvement in the adhesive characteristics of this mixture can be achieved by varying the ratio between the block copolymer and oil, leaving the ARB content unchanged.

### 3.3. The Effect of the Block Copolymer and Naphthenic Oil Ratio on the Properties of Adhesives

**Figure 6:**
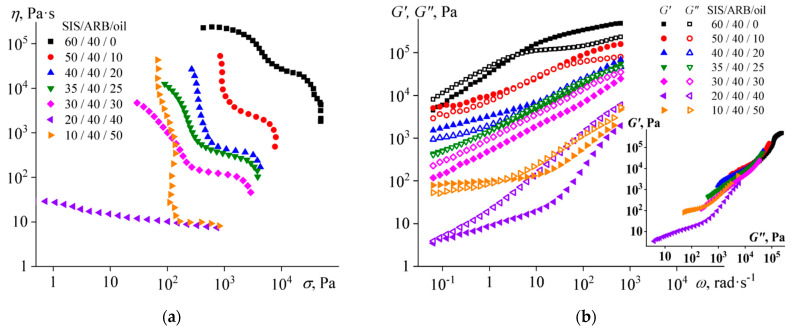
Flow curves (**a**) and frequency dependences of storage and loss moduli (**b**) for triple mixtures containing the same concentration of asphaltene/resin blend (ARB) and different ratios between styrene-isoprene-styrene triblock copolymer (SIS) and naphthenic oil at 120 °C. The insert shows the Cole–Cole plots.

The introduction of oil reduces the melt viscosity of the SIS/ARB mixture and makes its yield stress behavior more obvious (Figure 6a). The yield stress and viscosity decrease with increasing oil concentration, until the yield stress disappears at the oil content of 40%. However, as the oil concentration grows further up to 50%, the yield stress reappears, although the viscosity at high shear stresses remains almost equal to the viscosity of the mixture containing 40% of oil. In this case, the absence of yield stress at higher SIS concentration (20%) and lower oil content (40%) means better solubility of ARB in SIS rather than in the oil. Thus, the yield stress behavior at the high oil content can be associated with a decrease in the solubility of ARB and the appearance of the dispersed phase, which forms a spatial microstructure.

The viscoelasticity of the melt changes with increasing oil concentration in a similar way (Figure 6b). The behavior of the SIS/ARB mixture corresponds to the behavior of the polymer melt: a terminal zone is observed in the low-frequency region (*G*” > *G*’), which is replaced by a rubber-like state region (*G*’ > *G*”) as the frequency increases above 4 rad/s. The addition of 10–20% of oil reduces the values of both moduli, and the loss modulus decreases more intensively. As a result, the storage modulus exceeds the loss one in the entire frequency region, i.e., the mixture exhibits a solid-like behavior. It can be assumed that the oil penetrates mainly into the polyisoprene microphase, since otherwise if the oil penetrated significantly into the polystyrene blocks, it would lead to the formation of an ordinary polymer solution without yield stress and solid-like behavior. In turn, the saturation of polyisoprene blocks with the oil leads to a significant decrease in the loss modulus and viscosity. In this case, the polystyrene microphase is less susceptible to plasticization by oil, and therefore the storage modulus and the yield stress of the mixture are reduced less strongly.

The introduction of 30–40% of oil further reduces the moduli, and a liquid-like behavior in the entire frequency region becomes characteristic of the mixture. Moreover, when the content of the oil is 40%, the mixture has no yield stress. It can be concluded that there is either suppression of microphase separation or microphase inversion at this oil content, because of which the polyisoprene microphase becomes a continuous phase. The latter can occur if the oil predominantly penetrates into the polyisoprene microphase, causing its volume fraction to increase and start to exceed the volume fraction of the rigid polystyrene microphase. In any case, the yield stress and solid-like behavior at low frequencies return when the oil content is 50%, indicating the appearance of a new structural network in the sample volume at this oil content. Since the SIS content is as low as 10%, it can be assumed that this network does not consist of polystyrene microphase but rather of asphaltene particles remaining in the undissolved state due to the significantly higher ARB content compared to the SIS content.

**Figure 7:**
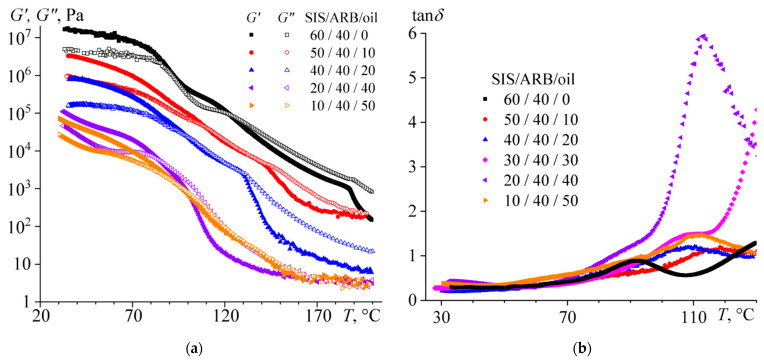
Temperature dependences of (**a**) storage and loss moduli and (**b**) loss tangent for triple mixtures containing the same concentration of asphaltene/resin blend (ARB) and different ratio between styrene-isoprene-styrene triblock copolymer (SIS) and naphthenic oil.

As for the temperature dependencies of rheological properties, the addition of 10–30% of oil reduces the storage and loss moduli in the entire temperature range and shifts the liquid–solid transition point towards lower temperatures (Figure 7a). Moreover, an extended temperature region appears on the temperature dependences of the storage and loss moduli, where both moduli take very similar values. This can be related to the expansion of the relaxation time spectrum of the mixture, which can be due to the partial miscibility of the polystyrene and polyisoprene blocks, i.e., suppression of microphase separation of the mixture by oil.

The introduction of 40% of oil leads to an even greater drop of the moduli, which start to depend more strongly on frequency even at low temperatures. This could be due to a decrease in the glass transition temperature of the polystyrene blocks, but this is not the case. When the oil is added, the shift of the loss tangent maximum towards higher temperatures can be found on the temperature dependences of the loss tangent (Figure 7b). It turns out that the introduction of oil does not plasticize the polystyrene blocks but reduces the plasticization, possibly due to the extraction of ARB from the polystyrene microphase. In addition, it can be assumed that the oil does not plasticize the rigid blocks of polystyrene but reduces their content because of suppression of microphase separation. The latter is supported by sharp multiple growths in the absolute value of the loss tangent when the content of the oil is 40%. As a result, the mixture exhibits liquid-like behavior down to lower temperatures and should be more suitable for the role of PSA. Meanwhile, the introduction of 50% of oil does not cause an increase in the loss tangent, but conversely *G*’ and *G*” become comparable in a very wide temperature range, i.e., some microstructure is formed in the mixture again. Most likely, this microstructure is generated by ARB, which forms a dispersed phase structured in the continuous medium of low-concentrated SIS solution in the oil.

**Figure 8:**
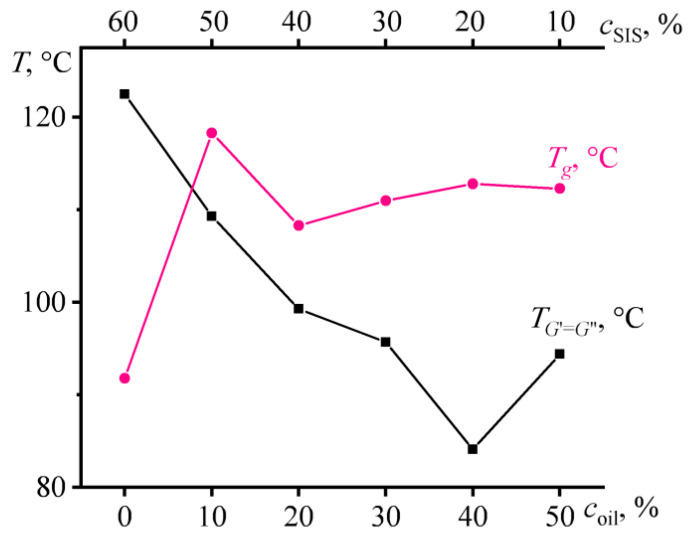
Concentration dependences of glass transition temperature and liquid–solid transition point for mixtures with different concentrations of naphthenic oil and styrene-isoprene-styrene triblock copolymer, while the concentration of asphaltene/resin blend (ARB) remains unchanged and equal to 40%.

The change in the glass and liquid–solid transition temperatures with varying the SIS/oil ratio is shown in Figure 8. Up to its 40% content, the oil effectively and monotonously reduces the temperature of the appearance of solid-like behavior. In contrast, the glass transition temperature passes through a maximum corresponding to 10% oil content and reaches a constant value at higher oil concentrations. Thus, the oil causes a redistribution of ARB between the microphases of polystyrene and polyisoprene in the direction of saturation of polyisoprene with ARB and reduces the volume fraction of the rigid polystyrene blocks. In other words, the oil predominantly dissolves in the polyisoprene microphase, increases its volume fraction, and thereby causes even greater overall solubility of ARB in this microphase due to its larger volume.

**Figure 9:**
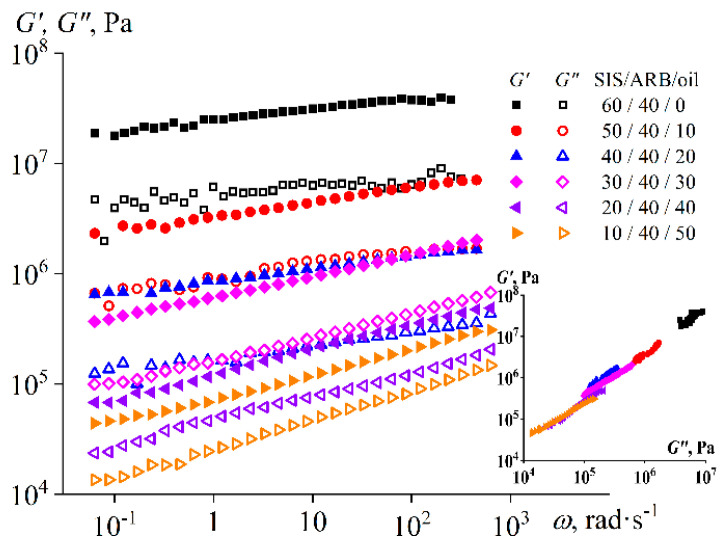
Frequency dependences of storage and loss moduli for triple mixtures containing the same concentration of asphaltene/resin blend (ARB) and different ratios between styrene-isoprene-styrene triblock copolymer (SIS) and naphthenic oil at 25 °C. The insert shows the Cole–Cole plots.

As a result, the rigidity of the mixture at 25 °C decreases uniformly with increasing oil concentration, but the samples retain their solid-like behavior even at concentrations of SIS and oil equal to 10% and 50%, respectively (Figure 9). Interestingly, all mixtures have the same microstructure at 25 °C according to the Cole–Cole plots, since they all approximately lie on a single line (the insert in Figure 9). At the same time, this was not the case at 120 °C: the Cole–Cole plots did not overlap (the insert in Figure 6b). The explanation may be that the samples were in different stages of microphase separation and glass transition at 120 °C, whereas at 25 °C the microphase separation was completed and so was the glass transition of the polystyrene blocks.

The addition of oil to the binary SIS/ARB blend monotonically reduces the cohesive strength of the blend and simultaneously the shear strength of the adhesive joints when the blend is used as HMA (Table 3 and its note containing the statistical analysis). Although the glued surfaces remained clean after the destruction of the adhesive joints, this indicates a mixed cohesive-adhesive nature of their failure. At the same time, the pull-off strength of the HMA joints passes through a maximum corresponding to the compositions with 10–20% of oil (*p* < 0.01). It is possible that the improvement in the apparent adhesive strength is associated with the decrease in the viscosity of the melt when oil is introduced into it (see Figure 6a), which allows HMA to better fill surface roughness. However, a further increase in the oil content causes a drop in cohesive strength (*p* < 0.01), a transition from adhesive to adhesive-cohesive nature of the destruction of adhesive joints, and a decrease in the apparent adhesive strength (*p* < 0.05).

If we are to consider the mixtures as PSA (Table 3), then this function appears in full when the content of oil is at least 40%. According to the Dahlquist criterion, the storage modulus of PSA should be below 3 × 10^5^ Pa in order to form a good adhesive joint [126,127]. This is the case when the oil content is more than 30%: the storage modulus is below the specified value (see Figure 9) and the apparent adhesive strength is high. At the same time, the mixture with 40% of oil demonstrates the best performance, having the most pronounced maximum of loss tangent (Figure 7b). Although the maximum of loss tangent occurs at a temperature of 112 °C, it has a high absolute value and an extended temperature width. In addition, the glass transition is a relaxation process. In other words, the maximum loss tangent at 112 °C is observed at a high angular frequency of deformation, at which these temperature dependences of loss tangent were obtained: at 6.28 rad/s which corresponds to an exposure time of 0.16 s. In the case of longer exposure times (such as when forming an adhesive joint for 10 s), the maximum loss tangent shifts towards lower temperatures. This means that an adhesive that does not stick well in a short exposure time can still stick if pressed for a longer time.

Thus, if considering the mixture containing 40% of ARB along with equal amounts of oil and SIS as a base, it is necessary to increase the SIS content to 50% and reduce the oil content to 10% in order to obtain a pure HMA that is not tacky at 25 °C. However, if it is desired to make a PSA, then the SIS content should conversely be reduced to 20%, increasing the oil concentration to 40%. Moreover, the resulting mixture can act as HMPSA. When using this mixture at high temperatures, the adhesive joint is an order of magnitude stronger (960 kPa vs. 103 kPa, see Table 3), although it does not reach the strength of the joints formed by HMAs (up to 2220 kPa). At the same time, the viscosity of this mixture in the melt is about 300 times lower than the viscosity of HMAs. This opens up completely different possibilities for using the mixture at elevated temperatures, for example, for easy impregnation of porous or dispersed objects or simple application to wide substrates without the use of special equipment operating at high pressures.

## 4. Discussion

Asphaltene/resin blend can be used as a modifier for poly(styrene-block-isoprene-block-styrene) triblock copolymer in order to turn it into an adhesive. In this case, the resins act as plasticizers of the block copolymer, partially suppressing its microphase separation and giving it tackiness. Plasticization is most effective when the ARB content is low, which leads to a significant decrease in the effective viscosity of the SIS melt. At the same time, asphaltenes act more like reinforcing particles, which are most effective when the ARB content is high. The nonlinearity of the change in the SIS properties when the ARB concentration is varied leads to the fact that as the ARB content increases, there is a transition from plasticization of the SIS to its hardening. This effect can be attributed to the limited miscibility of ARB with the block copolymer, which results in the formation of a separate microphase at high ARB concentrations. Meanwhile, ARB does not plasticize SIS sufficiently strongly, and the additional introduction of naphthenic oil is necessary to achieve better adhesion performance. In this case, it is reasonable to use ARB in high concentration to provide the effect of reinforcing the polymer with asphaltenes. In turn, the oil allows for more fine-tuning of the adhesive properties of the mixture, enabling the transition from conventional hot-melt adhesives to hot-melt pressure-sensitive adhesives as their concentration increases.

A practical question is how effective the ARB is as a tackifier. The standard tackifiers for SIS are hydrocarbon resins, which differ in chemical structure and molecular weight [10]. The use of commercial hydrocarbon resins (Regalite R9100, Escorez 5380) to modify the same SIS yields a hot melt adhesive with *τ*_s,HMA_ = 2070 kPa and *τ*_t,HMA_ = 390 kPa as the highest adhesive indicators [81]. The alternative use of indene-coumarone resin to produce HMA from SIS also results in comparable pull-off adhesion strength (*τ*_t,HMA_ = 370 kPa). In our case, the best HMA in terms of combined adhesive indicators is achieved at an SIS/ARB/oil ratio of 50/40/10, which gives *τ*_s,HMA_ = 980 kPa and *τ*_t,HMA_ = 1990 kPa (Table 3). In this regard, ARB stands behind conventional resins in terms of improving the shear strength of adhesive bonds but is several times superior in terms of improving the adhesion strength upon pull-off. As for pressure sensitivity, PSAs based on SIS and commercial hydrocarbon resin (Regalite R9100) under comparable conditions show *τ*_p,PSA_ = 650 N/m and *τ*_t,PSA_ = 400 kPa [128,129]. In our case, the best set of pressure-sensitive adhesive properties results from an SIS/ARB/oil ratio of 20/40/40, leading to *τ*_p,PSA_ = 600 N/m, and *τ*_t,PSA_ = 103 kPa. In this way, ARB provides less tackiness than hydrocarbon resin, but still maintains the formed adhesion bond well upon peeling. In other words, ARB is better suited to obtaining HMAs rather than PSAs. On this basis, ARB can generally be considered a low-cost alternative to commercial resins for adhesives designed for non-critical applications that do not require high specific adhesive strength. By way of example, this can be the bonding of wood chips for furniture production, which would allow the useful disposal of asphaltenes and resins remaining after deasphalting or distillation of heavy crude oils.

## 5. Conclusions

The investigation of rheological and adhesive properties of triple mixtures containing styrene-isoprene-styrene triblock copolymer (SIS), asphaltene/resin blend (ARB), and naphthenic oil has revealed the following:

− Both ARB and the oil do not disturb the microphase separation of the block copolymer and dissolve predominantly in the microphase of the polyisoprene blocks.
− The oil seems to be unlimitedly soluble in the block copolymer, whereas ARB dissolves up to a concentration of about 20% and then forms a separate phase. In addition, ARB and the oil are not fully miscible, which becomes apparent when the SIS content in the mixture is less than 20%.
− Dissolution of ARB and the oil increases the volume fraction of the polyisoprene microphase, which leads to a decrease in the viscosity of SIS and gives it the ability of irreversible deformation at very low frequencies (long observation times), thus causing stickiness. At the same time, the preservation of microphase separation ensures the dominance of elasticity at normal observation times, thus providing the strength of the formed adhesive joints.
− An increase in ARB concentration improves the cohesive and adhesive characteristics of the mixture, while an increase in the oil content gradually shifts the mixture from being a hot melt to being a pressure-sensitive adhesive with a simultaneous decline in the cohesive and adhesive performance. In this respect, the role of ARB is as both a tackifier and a filler, while the role of the oil is to be a plasticizer.

## Figures and Tables

**Table 1:** Strength and adhesion characteristics of styrene-isoprene-styrene triblock copolymer and its binary mixtures containing 40 wt% of naphthenic oil or asphaltene/resin blend (ARB).

Modifier	*τ*_c_, MPa	*τ*_s,HMA_, kPa	*τ*_t,HMA_, kPa	*τ*_p,PSA_, N m^−1^	*τ*_t,PSA_, kPa
-	4.2 ± 0.8	1300 ± 80	890 ± 350	0	0
ARB	6.5 ± 1.4 ^1,b^	1350 ± 230 ^d^	720 ± 340 ^d^	52 ± 11 ^b^	8 ± 13 ^d^
Oil	1.4 ± 0.1 ^a^	490 ± 55 ^a^	1100 ± 120 ^d^	3.5 ± 3.0 ^d^	35 ± 10 ^b^

^1^ The letter after the experimental data shows the *p*-value—the probability that adding a modifier did not actually affect this parameter: ^a^ *p* < 0.01 (good evidence that it does not), ^b^ 0.01 < *p* < 0.05 (moderate evidence), ^c^ 0.05 < *p* < 0.1 (weak evidence), ^d^ *p* > 0.1 (no evidence) [122].

**Table 2:** Strength and adhesion characteristics of the mixtures with different concentration of asphaltene/resin blend (ARB) and an equal ratio between styrene-isoprene-styrene triblock copolymer (SIS) and naphthenic oil.

SIS/ARB/Oil	*τ*_c_, MPa	*τ*_s,HMA_, kPa	*τ*_t,HMA_, kPa	*τ*_p,PSA_, N m^−1^	*τ*_t,PSA_, kPa
50/0/50	1.0 ± 0.2	2 ± 5	260 ± 30	0.1 ± 0.2	42 ± 5
45/10/45	1.0 ± 0.1 ^1,d^	9 ± 4 ^a^	700 ± 660 ^d^	240 ± 10 ^a^	50 ± 30 ^d^
40/20/40	0.6 ± 0.1 ^b^	420 ± 180 ^b^	700 ± 400 ^d^	490 ± 40 ^a^	60 ± 20 ^d^
35/30/35	0.7 ± 0.1 ^b^	590 ± 150 ^b^	600 ± 100 ^b^	710 ± 20 ^a^	52 ± 6 ^a^
30/40/30	1.0 ± 0.1 ^d^	750 ± 200 ^b^	1200 ± 300 ^b^	660 ± 150 ^a^	24 ± 11 ^b^

^1^ The letter after the experimental data shows the *p*-value—the probability that adding ARB did not actually affect this parameter: ^a^ *p* < 0.01 (good evidence that it does not), ^b^ 0.01 < *p* < 0.05 (moderate evidence), ^c^ 0.05 < *p* < 0.1 (weak evidence), ^d^ *p* > 0.1 (no evidence).

**Table 3:** Strength and adhesion characteristics of the mixtures with different ratios between styrene-isoprene-styrene triblock copolymer (SIS) and naphthenic oil and the same concentration of asphaltene/resin blend (ARB).

SIS/ARB/Oil	*τ*_c_, MPa	*τ*_s,HMA_, kPa	*τ*_t,HMA_, kPa	*τ*_p,PSA_, N m^−1^	*τ*_t,PSA_, kPa
60/40/0	4.2 ± 0.4 ^1,a^	1350 ± 230 ^a^	720 ± 340 ^a^	52 ± 11 ^b^	8 ± 13 ^a^
50/40/10	2.0 ± 0.3 ^b^	980 ± 140 ^a^	1990 ± 270 ^a^	12 ± 1 ^b^	11 ± 4 ^c^
40/40/20	1.70 ± 0.02 ^a^	780 ± 210 ^b^	2220 ± 280 ^a^	53 ± 3 ^b^	20 ± 11 ^d^
35/40/25	1.1 ± 0.4 ^d^	820 ± 90 ^d^	1710 ± 260 ^a^	160 ± 20 ^b^	22 ± 2 ^d^
30/40/30	1.0 ± 0.1	750 ± 200	1200 ± 300	660 ± 150	24 ± 11
20/40/40	0.40 ± 0.05 ^a^	250 ± 50 ^b^	960 ± 170 ^c^	600 ± 60 ^d^	103 ± 12 ^a^
10/40/50	0.20 ± 0.03 ^a^	110 ± 40 ^b^	900 ± 240 ^b^	440 ± 100 ^b^	94 ± 22 ^a^

^1^ The letter after the experimental data shows the *p*-value—the probability that the variation of the SIS/oil ratio did not actually affect this parameter as compared to the mixture containing the equal concentrations of SIS and oil: ^a^ *p* < 0.01 (good evidence that it does not), ^b^ 0.01 < *p* < 0.05 (moderate evidence), ^c^ 0.05 < *p* < 0.1 (weak evidence), ^d^ *p* > 0.1 (no evidence).

## Data Availability

The data presented in this study are available on request from the corresponding author.
